# Supplementary material for: Preparation and Characterization of Model Tire–Road Wear Particles
Source: Polymers (Basel). 2022 Apr 8;14(8):1512. doi: 10.3390/polym14081512 (PMC9027077; doi:10.3390/polym14081512)
Supplement: Supplementary file 1 [file polymers-14-01512-s001.zip › polymers-1655723-supplementary.pdf]

Preparation and characterization of model tire-road wear particles

Supplementary Information

Table S1. Formulation of the model TWP (phr).

Component	Content (phr)
NR	100
Carbon black	55
Curatives	9.7
Antidegradants	4
Processing oil	5

Table S2. EDS analysis results of the real MPs of 212- 500 μm (rMP1, rMP2, and rMP3) and the chloroform-treated TRWPs of 212- 500 μm (rTRWP1 and rTRWP2) collected on the road.

Element	Content (wt%)				
	rMP1	rMP1	rMP1	rTRWP1	rTRWP2
C	14.6	18.8	24.4	52.0	57.0
O	52.6	48.1	53.1	36.6	34.4
Na	0.4	5.9	---	---	0.3
Mg	---	1.4	0.7	0.3	---
Al	6.7	0.8	3.8	1.5	0.6
Si	18.6	20.6	12.7	4.3	5.2
S	---	---	---	0.5	0.6
K	6.6	---	1.2	0.5	0.2
Ca	---	4.0	0.6	1.7	0.7
Fe				1.6	0.6
Zn				0.8	0.3

Table S3. EDS analysis results of the model asphalt pavement wear particles (APP1 and APP2), the model concrete pavement wear particles (CPP1 and CPP2), and the model stone wear particles (SP1 and SP2) of 20 - 38 μm.

Element	Content (wt%)					
	APP1	APP2	CPP1	CPP2	SP1	SP2
C	45.5	32.1	15.5	11.2	11.4	9.3
O	40.6	46.1	53.0	50.8	48.7	52.8
Na	---	---	---	---	---	1.9
Mg	0.2	0.2	7.8	1.9	13.8	3.1
Al	0.8	0.6	0.5	1.5	11.1	6.6
Si	3.1	2.4	1.3	6.5	9.8	13.9
S	---	---	---	---	---	1.6
K	0.4	---	---	---	1.6	2.9
Ca	9.5	18.5	21.4	25.3	3.6	3.0
Ti	---	---			---	0.5
Fe	---	---	0.5	1.3	---	5.9

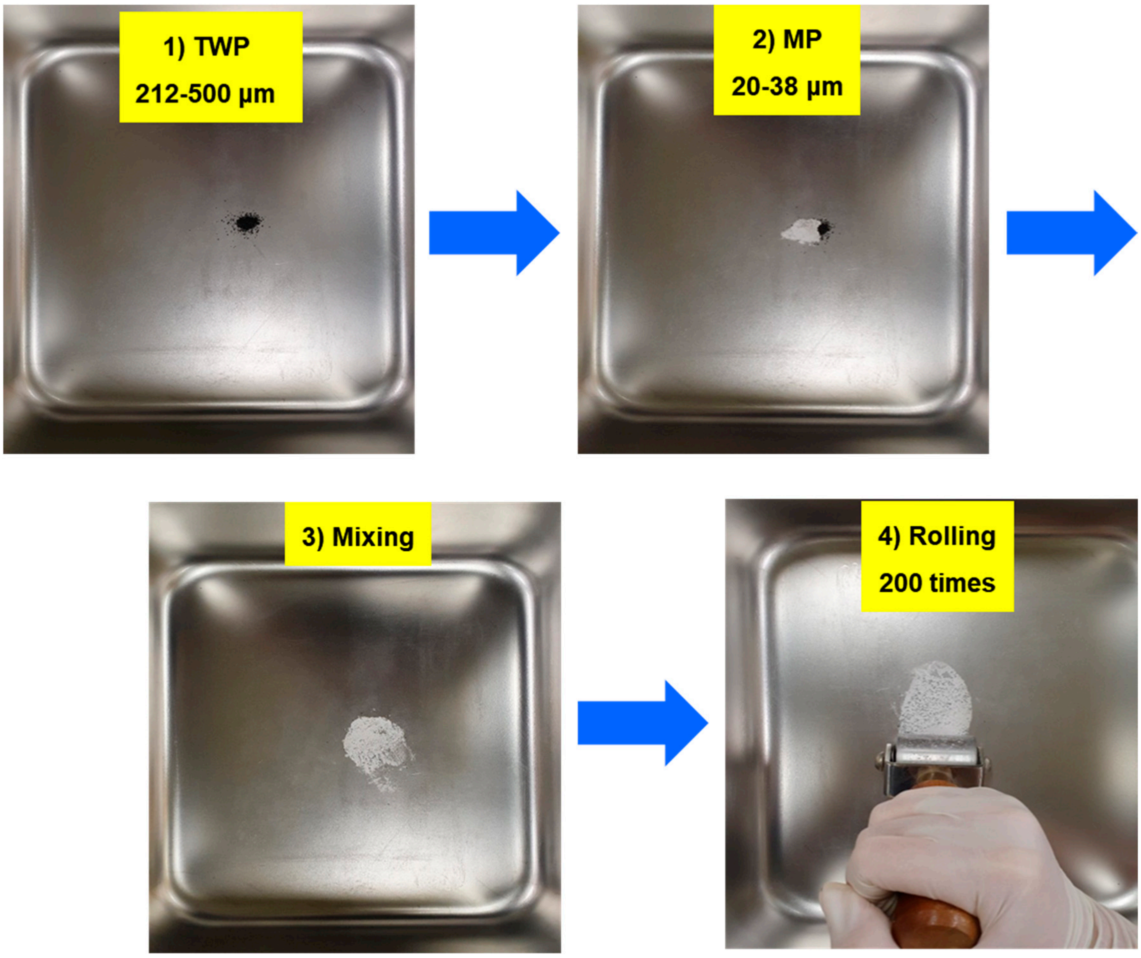

Figure S1. Preparation procedure of model TRWP.

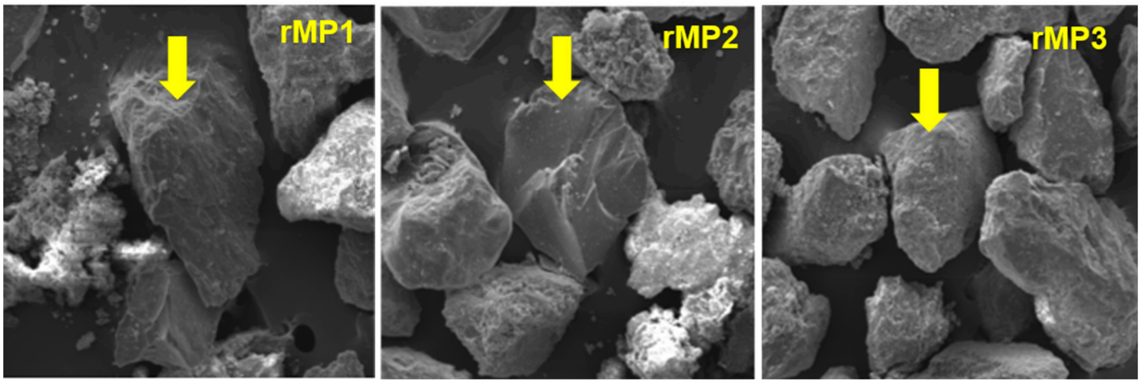

Figure S2. SEM images of the real MPs of 212 – 500 μm collected at the bus stop.

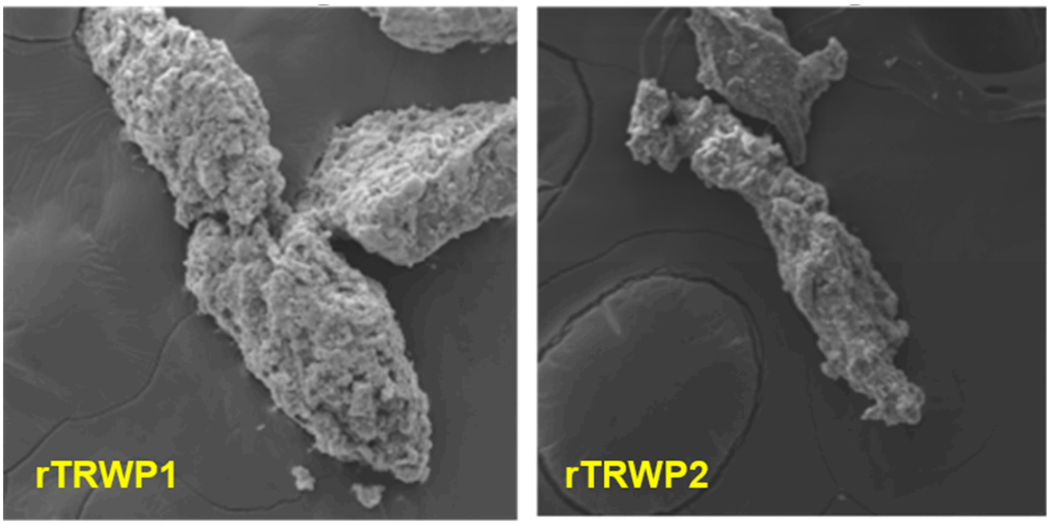

Figure S3. SEM images of the chloroform-treated real TRWPs of 212 – 500 μm collected at a bus stop.

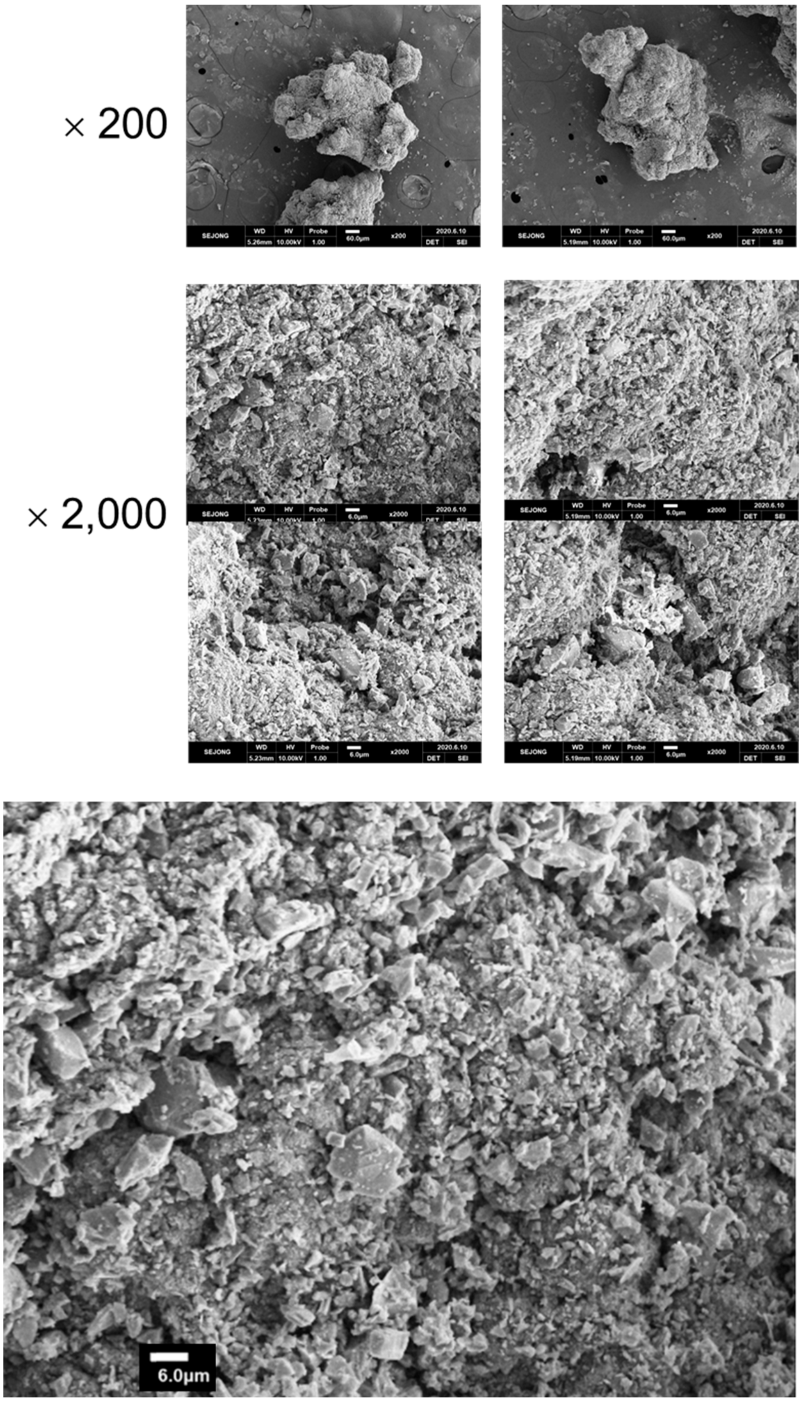

Figure S4. SEM images of the model TRWPs made of TWPs without treatment by the single step pressing procedure.

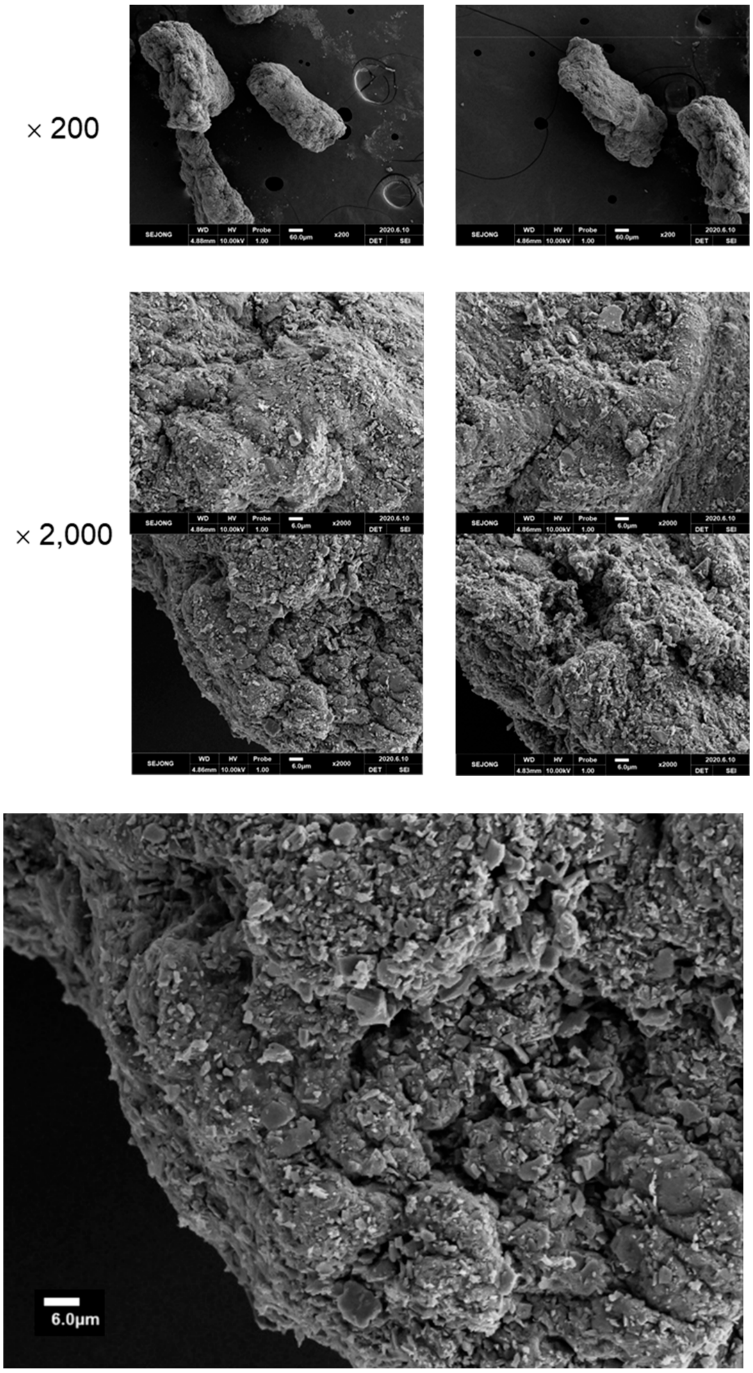

Figure S5. SEM images of the model TRWPs made of TWPs without treatment by the double step pressing procedure.

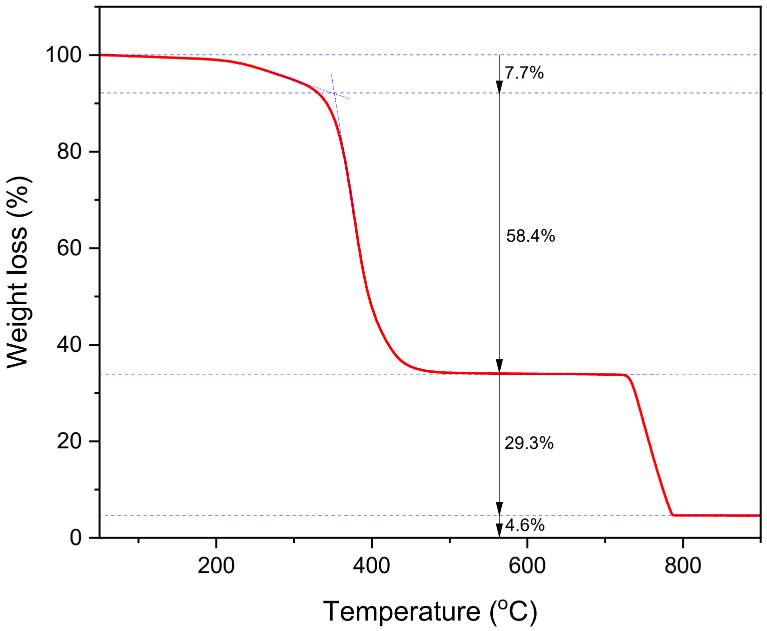

Figure S6. TGA thermogram of the model TWP.

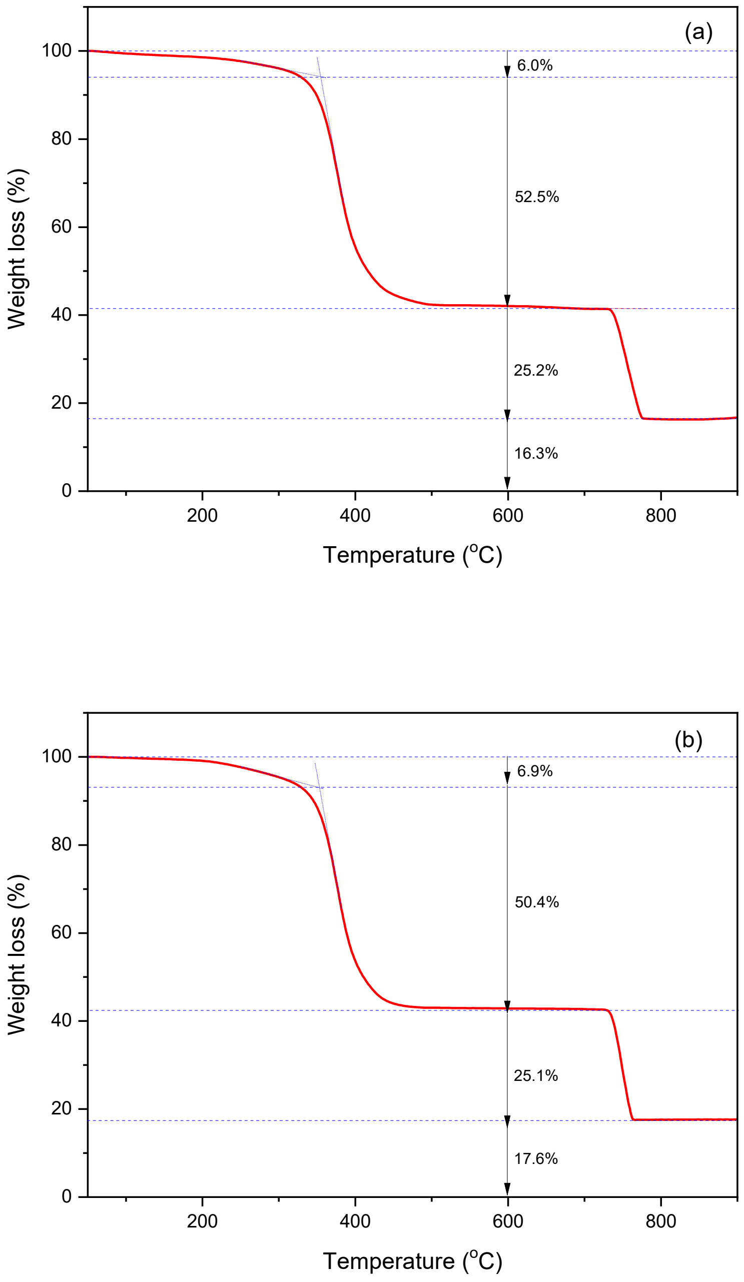

Figure S7. TGA thermograms of the model TRWPs made of TWPs without treatment by the single (a) and double (b) step pressing procedures.

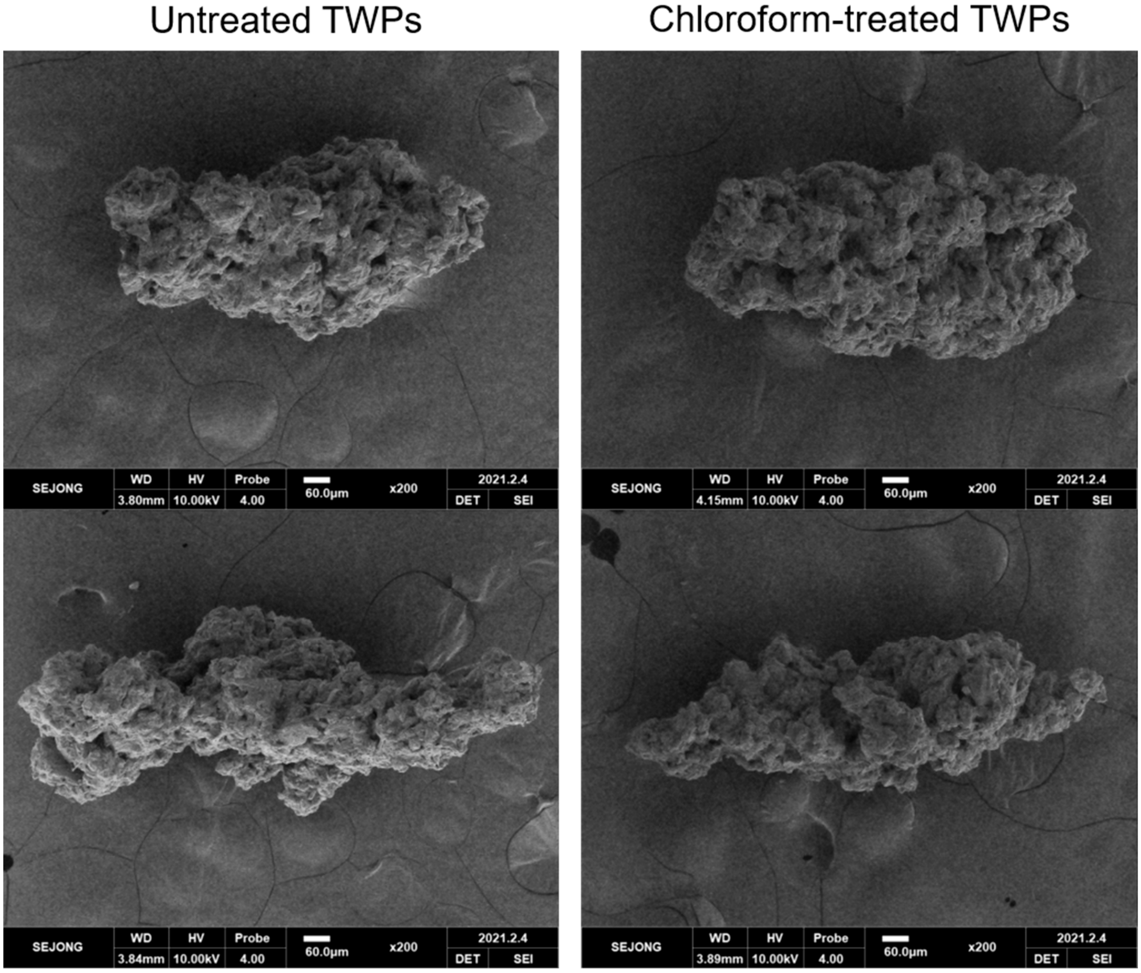

Figure S8. SEM images of the untreated and chloroform-treated TWPs.

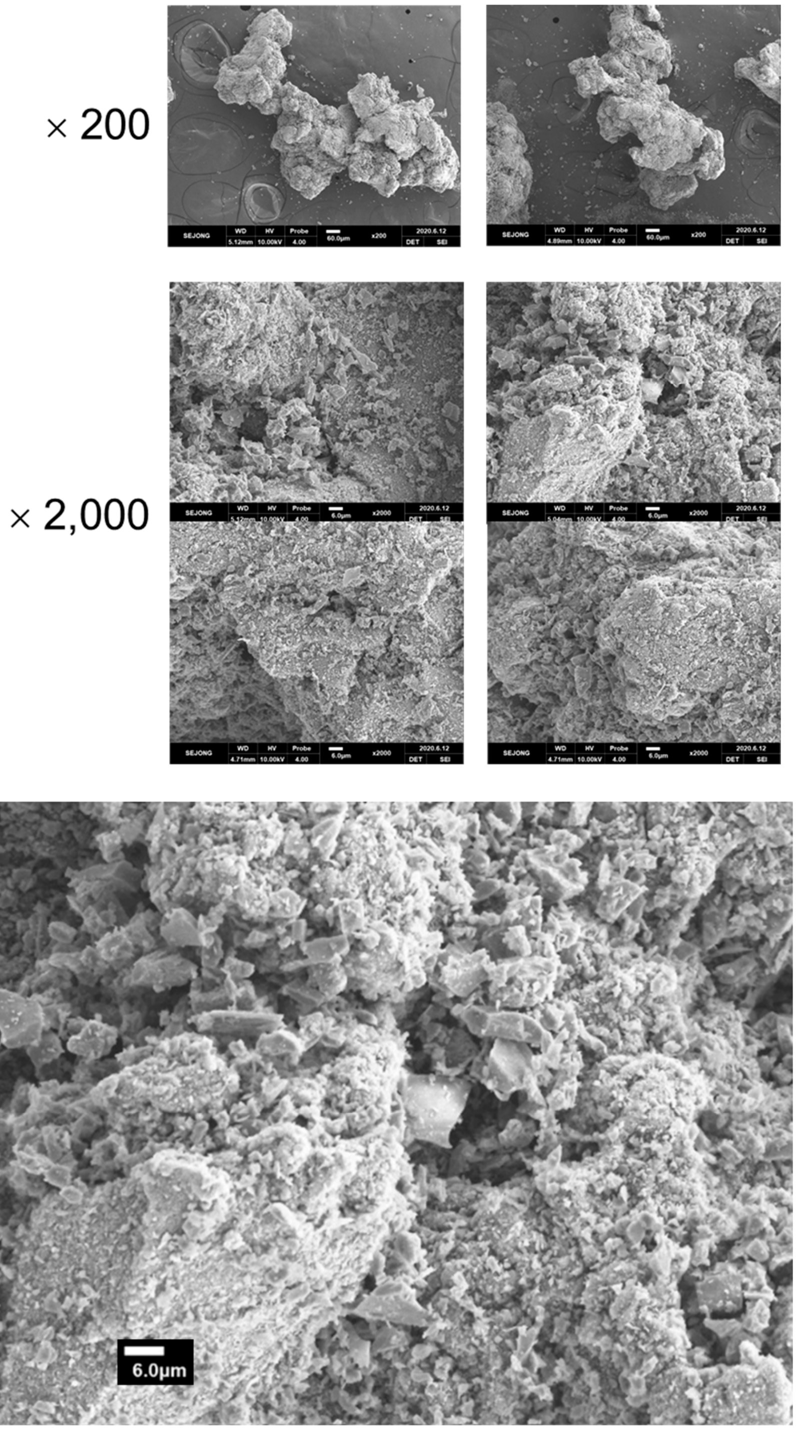

Figure S9. SEM images of the model TRWPs made of the chloroform-treated TWPs by the single step pressing procedure.

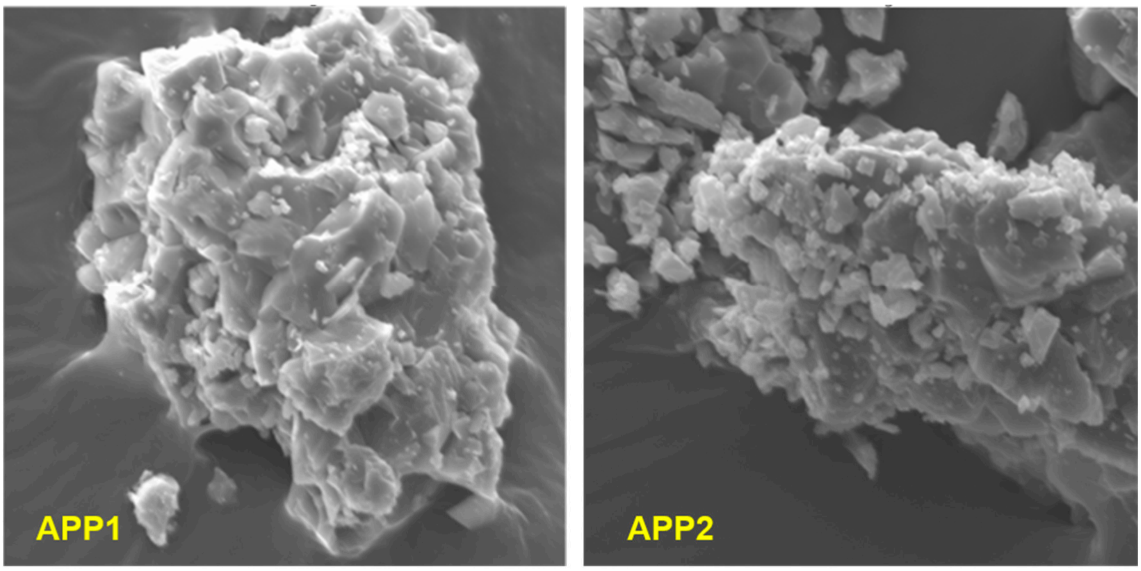

Figure S10. SEM images of the model asphalt pavement wear particles of 20 - 38 μm.

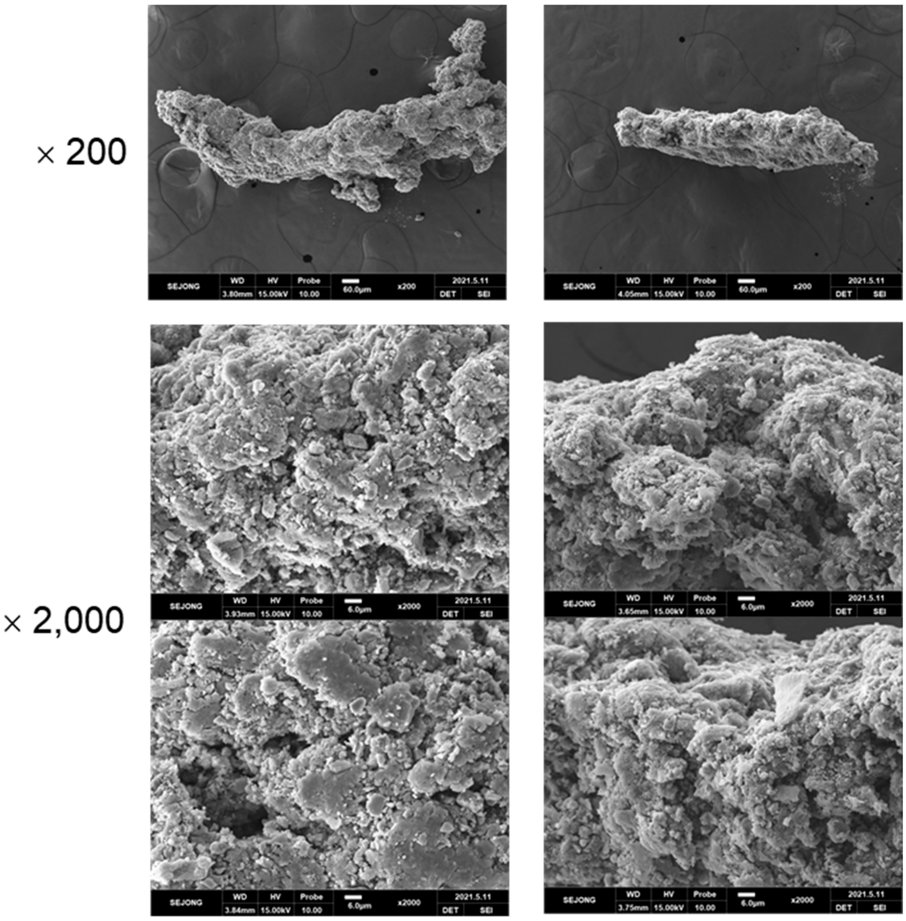

Figure S11. SEM images of the model TRWPs made of the untreated TWPs (212 - 500 μm) and the asphalt pavement wear particles (20 - 38 μm).

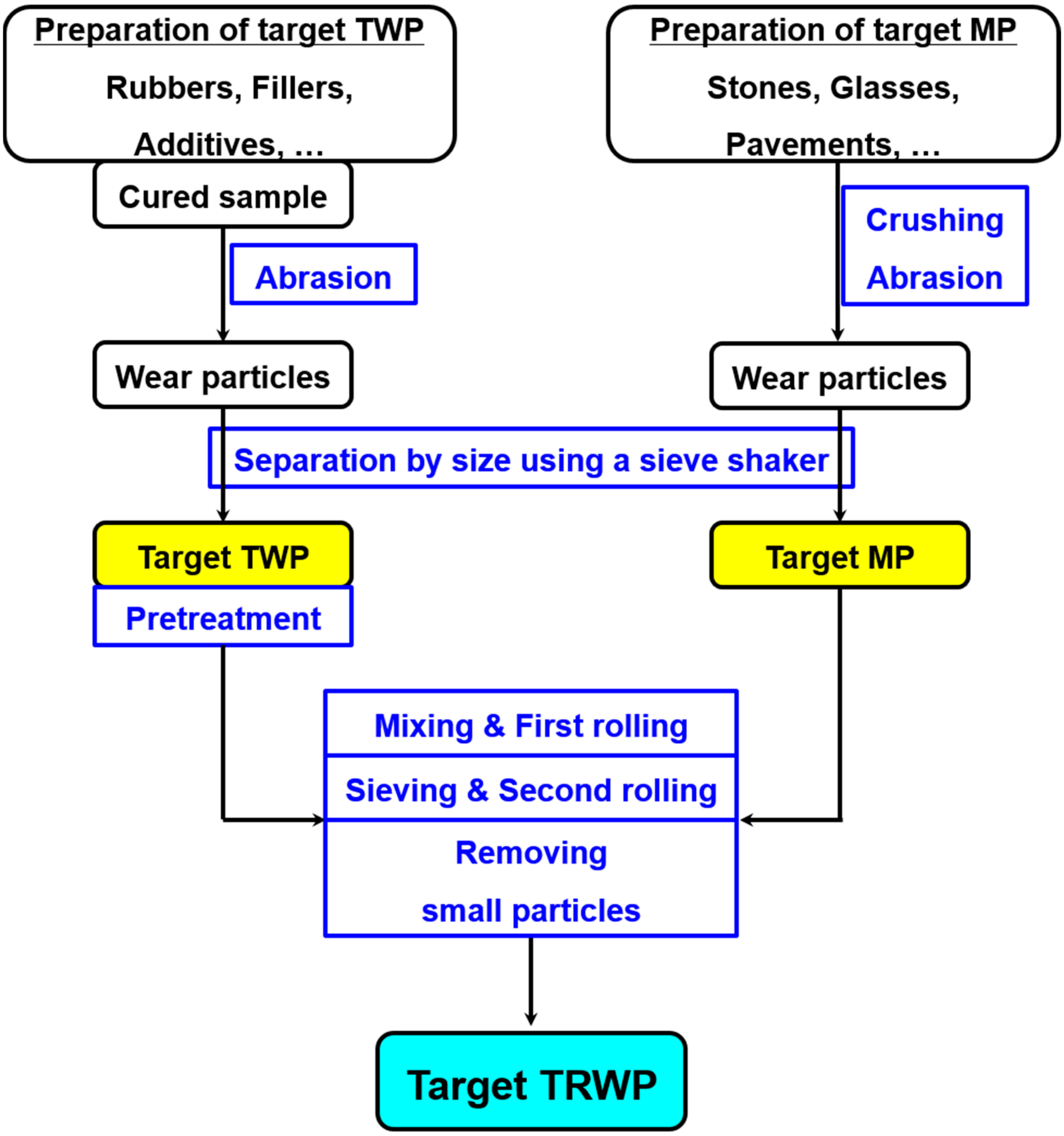

Figure S12. Preparation process of tailor-made TRWPs.

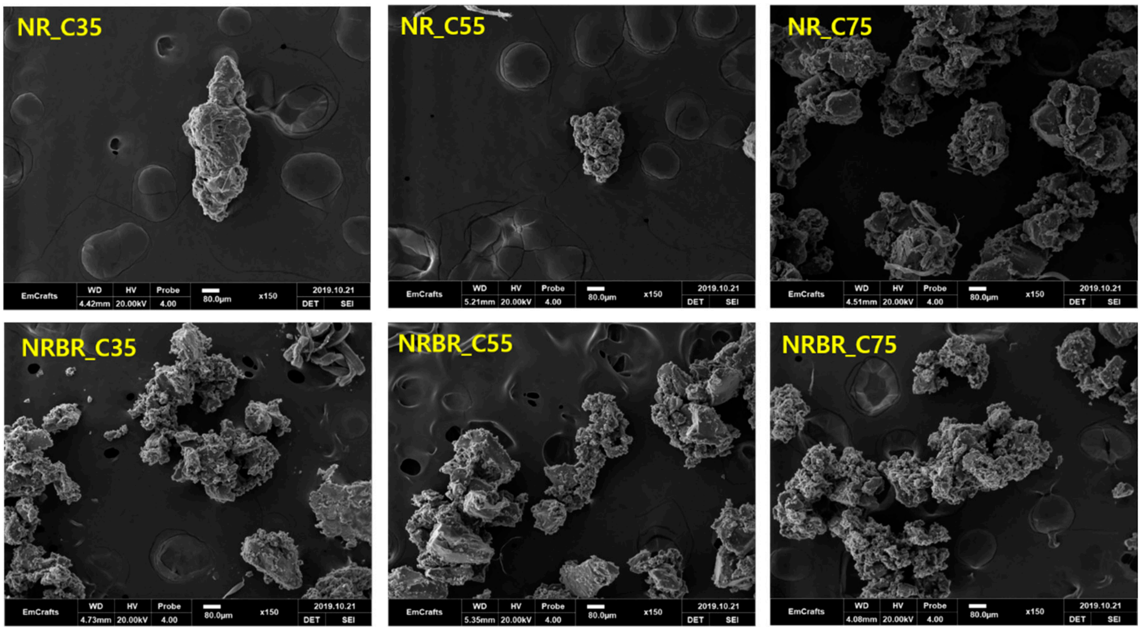

Figure S13. SEM images of various TWPs made by cryogenic crushing. The NR_C35, NR_C55, and NR_C75 samples are NR = 100 vulcanizates with carbon black contents of 35, 55, and 75 phr, respectively. The NRBR_C35, NRBR_C55, and NRBR_C75 samples are NR/BR = 80/20 vulcanizates with carbon black contents of 35, 55, and 75 phr, respectively.

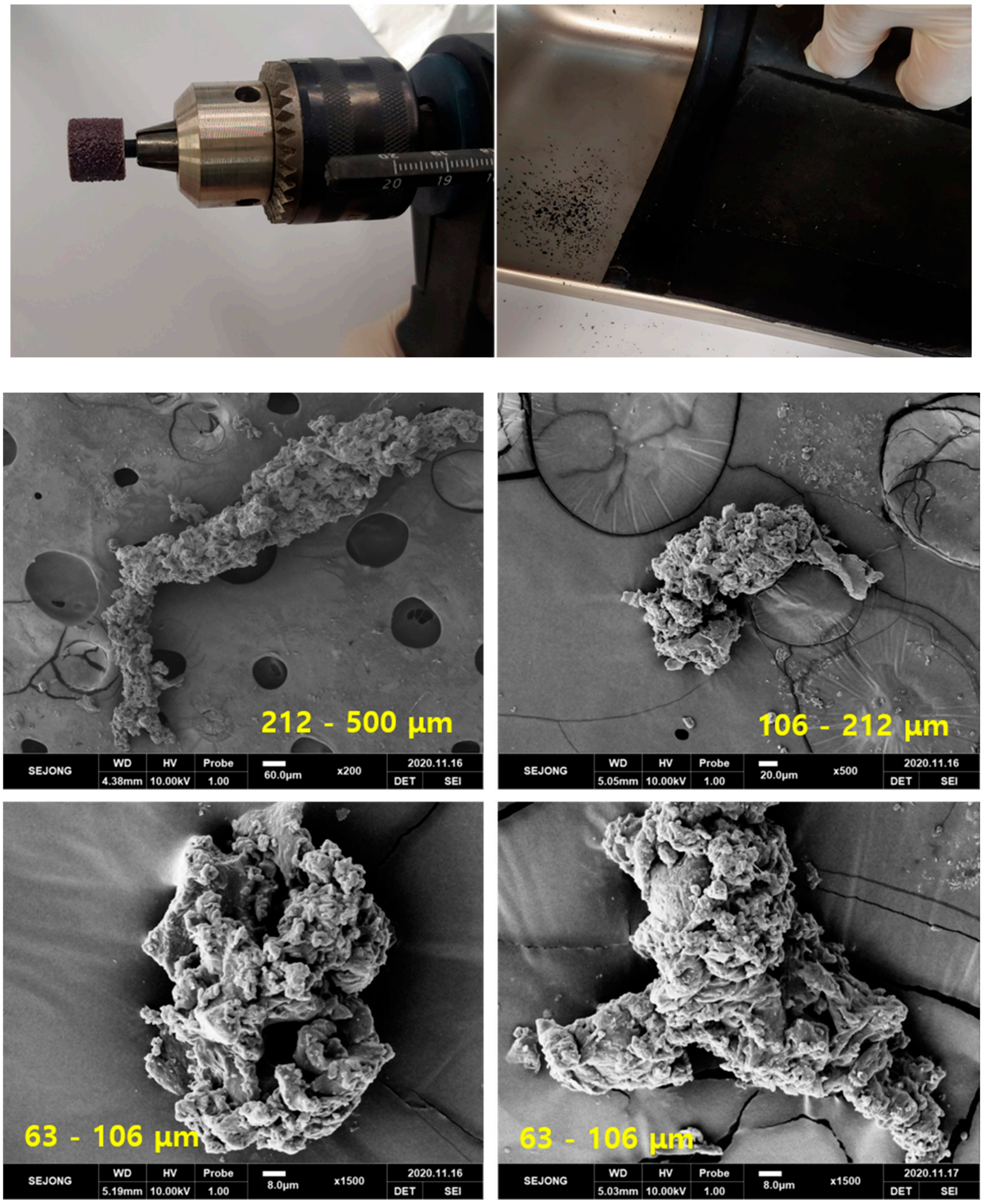

Figure S14. Photographs of preparation of TWPs using a hand drill and a tensile test specimen, and SEM images of the various SBR TWPs made using the hand drill method.

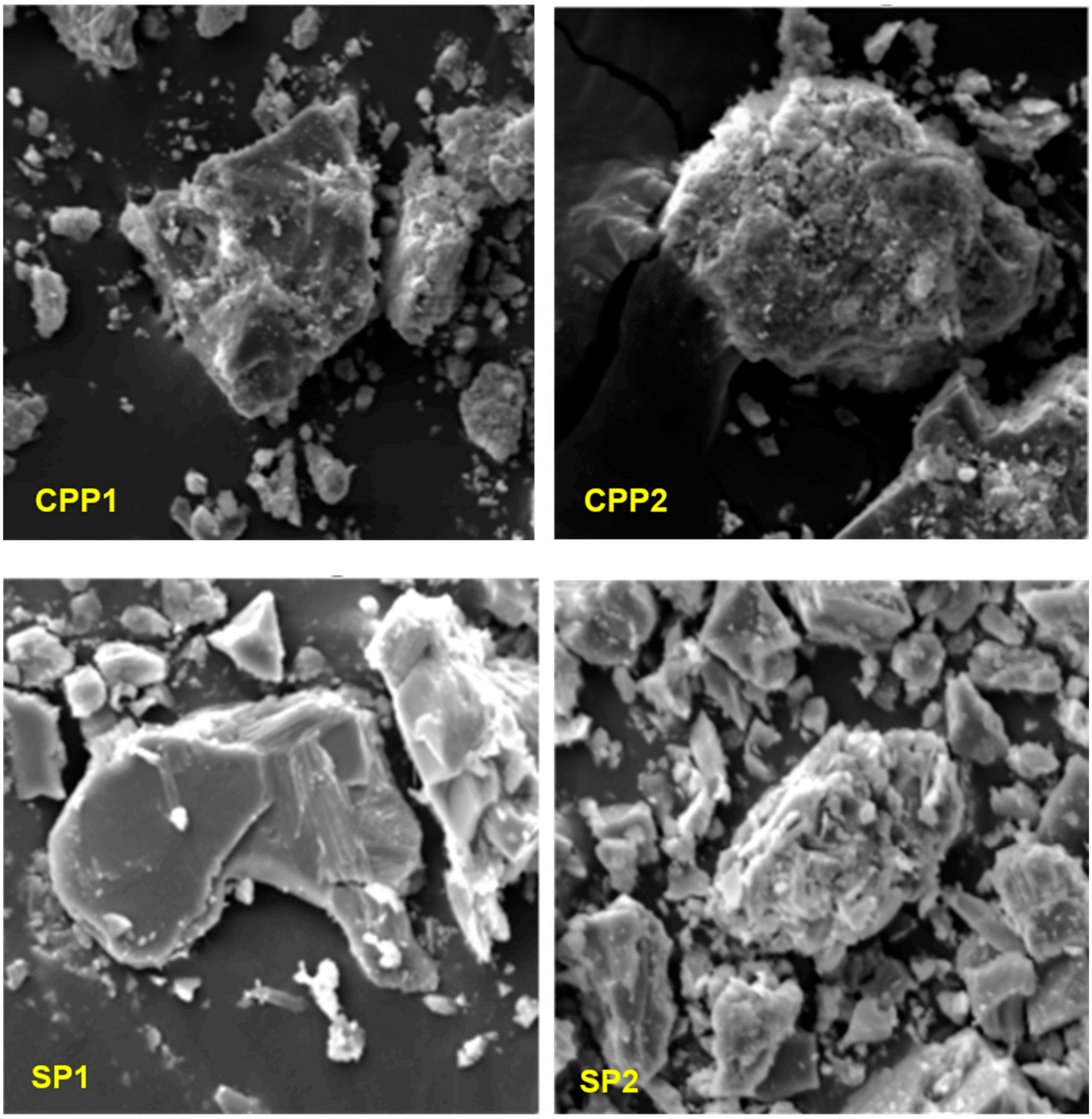

Figure S15. SEM images of the model concrete pavement (CPP1 and CPP2) and stone (SP1 and SP2) wear particles of 20 - 38 μm.
